# Decreasing resection rates for nonmetastatic gastric cancer in Europe and the United States

**DOI:** 10.1002/ctm2.203

**Published:** 2020-10-08

**Authors:** Lei Huang, Lina Jansen, Yesilda Balavarca, Rob H.A. Verhoeven, Jelle P. Ruurda, Liesbet Van Eycken, Harlinde De Schutter, Jan Johansson, Mats Lindblad, Tom B. Johannesen, Vesna Zadnik, Tina Žagar, Margit Mägi, Esther Bastiaannet, Sjoerd M. Lagarde, Cornelis J.H. van de Velde, Petra Schrotz‐King, Hermann Brenner

**Affiliations:** ^1^ Division of Clinical Epidemiology and Aging Research German Cancer Research Center (DKFZ) Heidelberg Germany; ^2^ Medical Faculty Heidelberg Heidelberg University Heidelberg Germany; ^3^ German Cancer Consortium (DKTK) German Cancer Research Center (DKFZ) Heidelberg Germany; ^4^ Division of Preventive Oncology German Cancer Research Center (DKFZ) and National Center for Tumor Diseases (NCT) Heidelberg Germany; ^5^ Department of Research Netherlands Comprehensive Cancer Organization (IKNL) Utrecht The Netherlands; ^6^ Department of Surgery Radboud University Medical Centre Nijmegen The Netherlands; ^7^ Department of Surgery University Medical Center Utrecht Utrecht The Netherlands; ^8^ Belgian Cancer Registry (BCR) Brussels Belgium; ^9^ Department of Esophageal and Gastric Surgery Lund University Hospital Lund Sweden; ^10^ Department of Clinical Science, Intervention, and Technology (CLINTEC), Division of Surgery Karolinska University Hospital Stockholm Sweden; ^11^ Registry Department The Cancer Registry of Norway (CRN) Oslo Norway; ^12^ Epidemiology and Cancer Registry Institute of Oncology Ljubljana Ljubljana Slovenia; ^13^ Estonian Cancer Registry National Institute for Health Development Tallinn Estonia; ^14^ Department of Surgical Oncology Leiden University Medical Center Leiden The Netherlands; ^15^ Department of Surgery Erasmus MC‐University Medical Centre Rotterdam Rotterdam The Netherlands

**Keywords:** gastric cancer, international population‐based study, patterns, policymaking and resource allocation, resection, trends, variation

## Abstract

**Background:**

Resection is the cornerstone of curative treatment for many nonmetastatic gastric cancers (GCs), but the population treatment patterns remains largely unknown. This large international population‐based study aimed at investigating the treatment patterns and trends for nonmetastatic GC in Europe and the United States and at exploring factors associated with resection.

**Methods:**

Data of patients with microscopically confirmed primary invasive GC without distant metastasis from the national cancer registries of the Netherlands, Belgium, Sweden, Norway, Slovenia, and Estonia and the US Surveillance, Epidemiology, and End Results (SEER)‐18 Program were retrieved. Age‐standardized treatment rates were computed and trends were evaluated using linear regression. Associations of resection with patient and tumor characteristics were analyzed using multivariable‐adjusted log‐binomial regression. Analysis was performed in each country respectively without pooling.

**Results:**

Together 65 707 nonmetastatic GC patients diagnosed in 2003‐2016 were analyzed. Age‐standardized resection rates significantly decreased over years in all countries (by 4‐24%). In 2013‐2014, rates varied greatly from 54 to 75%. Patients with increasing ages, cardia cancers, or cancers invading adjacent structure were significantly less frequently resected. Resection was further associated with sex, performance status, comorbidities, tumor histology, tumor size, hospital type, and hospital volume. Association patterns and strengths varied across countries. After multivariable adjustment, resection rates remained decreasing (prevalence ratio = 0.97‐0.995 per year), with decreasing trends consistently seen in various subgroups.

**Conclusions:**

Nonmetastatic GCs were less frequently resected in Europe and the United States in the early 21st century. Resection rates varied greatly across countries and appeared not to be optimal. Various factors associated with resection were revealed. Our findings can help to identify differences and possibly modifiable places in clinical practice and provide important novel references for designing effective population‐based GC management strategies.

- In Europe and the United States, nonmetastatic gastric cancers were less frequently resected in the early 21st century.
- Resection rates varied greatly across countries and appeared not optimal.
- Various factors associated with resection were revealed.
- Our findings identify differences and possibly modifiable places in clinical practice and provide important novel references for designing effective population‐based management strategies.

## 1 BACKGROUND

Worldwide approximately 1 034 000 patients are estimated to be newly diagnosed with gastric cancer (GC) and approximately 783 000 GC‐associated deaths are estimated to occur in 2018, making it the fifth most commonly diagnosed malignancy and the third leading cause of cancer‐related mortality.^1^ The majority of patients with early‐stage GCs can be cured, however, in Western countries many GC patients have advanced diseases at diagnosis.^2^ Adequate resection remains the cornerstone of potentially curative treatment which can assure long‐term survival for medically fit patients with resectable nonmetastatic GC.^3^, ^4^, ^5^ Notably, involvement of peristomach structures in nonmetastatic cancers might preclude resection.

GC is marked for the global variations in etiology, incidence, patient and tumor characteristics, management, and outcomes.^6^, ^7^ Being less prevalent, GC care has not been well investigated in Western countries, which potentially hampers survival improvement. Real‐world GC treatment patterns at the population level, which may be directly associated with the overall survival statistics, have remained largely unknown in most Western countries except the Netherlands.^8^, ^9^ Notably, the application of resection, which is the fundamental treatment for GC, has been rarely studied. International analyses of treatment patterns and trends could help to identify differences and possibly modifiable patterns in clinical practice, of potential relevance for guiding adequate health policymaking and resource allocation.

In this large international population‐based study, we investigated the application of resection for nonmetastatic GCs in Europe and the United States and explored the factors associated with resection.

## 2 METHODS

### 2.1 Patients

An extensive retrieval of nationwide population‐based registries was performed, and the selection of contacted European registries is shown in Supporting information Table S1. Individual‐level data of GC patients from national population‐based cancer registries of the Netherlands, Belgium, Sweden, Norway, Slovenia, and Estonia, and the US Surveillance, Epidemiology, and End Results (SEER)‐18 Program were finally included (Table 1).The SEER Program is an authoritative source for cancer statistics in the United States, and currently collects data on cancer incidence and survival from population‐based cancer registries covering approximately 35% of the US population.^10^ Registry characteristics have been described in detail previously,^11^ and the data were generally *of high quality*. This study was approved by the Ethics Committee of the Medical Faculty Heidelberg.

**TABLE 1:** General information on participating population‐based registries

				Excluded cases^1^						
Source	Country	Diagnosis period	Registered cases	DCO/autopsy	Not pathologically diagnosed	Not pathologically eligible^2^	Precancerous/*in situ*	Unknown metastasis	Metastatic	Analyzed cases
SEER‐18^3^	The US	Jan. 2004‐Dec. 2015	79 091	855 (1)	983 (1)	10 020 (13)	780 (1)	5344 (7)	23 280 (29)	37 829
NCR	Netherlands	Jan. 2005‐Dec. 2014	18346	48 (< 1)	309 (2)	78 (< 1)	343 (2)	711 (4)	7112 (39)	9745
BCR	Belgium	Jan.2004‐Dec. 2013	14122	NA	80 (1)	1670 (12)	23 (< 1)	3076 (22)	2805 (20)	6468
SCR	Sweden	Jan. 2006‐Aug. 2016	7909	NA	169 (2)	0 (0)	0 (0)	471 (6)	2783 (35)	4486
CRN	Norway	Jan. 2003‐Dec. 2014	6194	53 (1)	299 (5)	438 (7)	5 (< 1)	362 (6)	1779 (29)	3258
CRS	Slovenia	Jan. 2003‐Dec. 2013	5265	NA	241 (5)	231 (4)	9 (< 1)	236 (4)	1655 (31)	2893
ECR	Estonia	Jan. 2009‐Dec. 2014	2394	67 (3)	139 (6)	114 (5)	0 (0)	245 (10)	801 (33)	1028

^1^ Shown as *n* (percentage [%]).

^2^ Based on criteria shown in Supporting information Table S3. Preliminary case selection according to cancer histology had been performed by the national cancer registries of the Netherlands and Sweden.

^3^ Data of the year 2003 were not analyzed, as the TNM stage (version 6/7) information was unavailable.

SEER, Surveillance, Epidemiology, and End Results Program; NCR, Netherlands Cancer Registry; BCR, Belgian Cancer Registry; SCR, Swedish Cancer Registry; CRN, Cancer Registry of Norway; CRS, Cancer Registry of Slovenia; ECR, Estonian Cancer Registry; DCO, death certificate only; NA, not available.

Cancer topography and morphology followed the International Classification of Diseases for Oncology, Third Edition.^12^ Only patients with *microscopically confirmed* primary invasive malignancies of the stomach (C16) registered in 2003 through 2017 were selected. To focus on patients who may have potentially curative resections and for whom those resections would make a considerable difference to outcomes, analyses were restricted to cancers without distant metastasis. Both cardia and noncardia GCs were included. Individuals with noninvasive benign/premalignant/in situ tumors, non‐GC neoplasms, involving the stomach, gastrointestinal stromal tumors/sarcomas, neuroendocrine tumors/carcinoids, lymphomas, or germ‐cell neoplasms, were excluded (Supporting information Table S2). Cases diagnosed based on death certificate only (DCO)/autopsy were also excluded.

Data on patient (year of diagnosis, sex, and age), cancer (location, differentiation, histology, and stage), treatment (resection, chemotherapy, and radiotherapy), and follow‐up variables (survival time and status) (re)coded following a uniform data‐request sheet were obtained. Information on hospital type (the Netherlands, Belgium, and Sweden), hospital volume (the Netherlands, Belgium (for resected patients only), and Sweden), tumor size (the US), Eastern Cooperative Oncology Group Performance Status (ECOG‐PS) score (Belgium and Sweden), American Society of Anesthesiologists (ASA) score (Sweden), and comorbidities (Eindhoven, the Netherlands and Belgium) were only available in certain registries.

*Resection was defined as removal of the primary tumor irrespective of the type, extent, and radicality of excision and lymphadenectomy, and of the method, approach, procedure, and technique of management, and included open, minimally invasive, and endoscopic (only used for a small proportion of cancers with invasion limited to lamina propria/submucosa) resection*. Tumor local invasion, lymph node involvement, and distant metastasis were derived from the American Joint Committee on Cancer (AJCC)/Union for International Cancer Control (UICC) TNM staging, and were reclassified into categories consistent across the investigated period when the sixth/seventh edition was in effect.

### 2.2 Statistics

Data were analyzed and presented separately in each country without pooling. Patient age was categorized into four groups (<60, 60‐69, 70‐79, and ≥80 years). Age‐standardized treatment rates were calculated using the age distribution of the US patients, the largest group of patients analyzed, as the standard. Temporal trends of the standardized rates were assessed using linear regression, and rates over two‐calendar‐year periods were shown graphically. Subgroup analyses according to patient age and tumor location were further conducted, and age‐ and location‐specific rates in 2010 or later were graphically illustrated.

Multivariable log‐binomial regression models were constructed to investigate the associations of resection with patient and tumor characteristics with adjustment for year of diagnosis, sex, age group, tumor location, and histology in main analyses. Log‐binomial maximum likelihood prevalence ratios (PRs) were computed.^13^ Subgroup analyses according to age, location, histology, and invasion of adjacent structures were further conducted. Further sensitivity analyses were performed by limiting patients to those with tumor invasion beyond submucosa and/or with positive lymph nodes where endoscopic resection was rarely performed. Associations with additional variables (adjacent structure invasion, hospital type and volume, tumor size, ECOG‐PS and ASA scores, and comorbidities) were evaluated by adding them one by one into the main models in countries with available information. SAS software (v.9.4; Cary, NC) was used for analyses. Statistical significance was defined by two‐sided *P *< .05.

## 3 RESULTS

Overall 1 33 321 GC patients registered in the population‐based registries were initially included (Table 1). Patients with DCO/autopsy‐based diagnosis (1%), without microscopically confirmed or eligible pathology (11%), with noninvasive diseases (1%), without information on distant metastasis status (8%), and with distant metastasis (30%) were excluded. Exclusion of patients with unknown metastasis status affected overall resection rates by only 0‐3% units in the United States, the Netherlands, Sweden, Norway, and Slovenia, but markedly increased the resection rate in Belgium (from 51 to 61%), where the proportion of unknown metastasis was relatively high (22%; Supporting information Table S3). Finally 65 707 patients with nonmetastatic disease were analyzed. Characteristics of overall and resected nonmetastatic GC patients are shown in Table 2 and detailed in Supplementary Results.

**TABLE 2:** Demographic and clinical characteristics of total and resected gastric cancer patients without distant metastasis^1^

		The US, 2004–15		Netherlands, 2005‐14		Belgium, 2004‐13		Sweden, 2006‐16		Norway, 2003‐14		Slovenia, 2003‐13		Estonia, 2009‐14	
**Variable**	Category	Total	Resected	Total	Resected	Total	Resected	Total	Resected	Total	Resected	Total	Resected	Total	Resected
**n**		37 829	25 070 (66)	9745	6605 (68)	6468	5096 (79)	4486	2501 (56)	3258	2057 (63)	2893	2172 (75)	1028	807 (79)
**Sex**	Male	24 063 (64)	15 989 (64)	6287 (65)	4367 (66)	4274 (66)	3420 (67)	2821 (63)	1587 (63)	2036 (62)	1323 (64)	1821 (63)	1384 (64)	568 (55)	445 (55)
**Age at diagnosis**	Year; as continuous	69 ± 13	67 ± 13	71 ± 12	68 ± 12	70 ± 13	69 ± 12	72 ± 12	69 ± 11	72 ± 12	70 ± 12	69 ± 12	67 ± 12	68 ± 12	67 ± 12
**Age group**	< 60 years	8672 (23)	6603 (26)	1699 (17)	1423 (22)	1274 (20)	1110 (22)	714 (16)	506 (20)	520 (16)	379 (18)	642 (22)	567 (26)	223 (22)	194 (24)
	60‐69 years	8865 (23)	6559 (26)	2280 (23)	1815 (27)	1390 (22)	1191 (23)	1043 (23)	720 (29)	725 (22)	530 (26)	662 (23)	553 (25)	273 (27)	222 (28)
	70‐79 years	10 528 (28)	7286 (29)	3144 (32)	2230 (34)	2122 (33)	1733 (34)	1394 (31)	831 (33)	968 (30)	664 (32)	1000 (35)	770 (35)	332 (32)	261 (32)
	≥80 years	9764 (26)	4622 (18)	2622 (27)	1137 (17)	1682 (26)	1062 (21)	1335 (30)	444 (18)	1045 (32)	484 (24)	589 (20)	282 (13)	200 (19)	130 (16)
**Tumor location** ^2^	Gastric cardia	12 731 (48)	7387 (42)	2630 (37)	1622 (33)	2024 (55)	1520 (54)	1387 (39)	737 (35)	857 (39)	480 (32)	461 (27)	314 (23)	96 (12)	70 (11)
	Gastric fundus/body	4461 (17)	2992 (17)	1576 (22)	1132 (23)	483 (13)	368 (13)	1018 (28)	611 (29)	486 (22)	322 (22)	459(27)	414 (30)	423 (52)	342 (53)
	Gastric antrum/pylorus	9578 (36)	7208 (41)	2957 (41)	2236 (45)	1152 (32)	939 (33)	1196 (33)	762 (36)	865 (39)	678 (46)	779 (46)	654 (47)	291 (36)	237 (37)
	Other	11 059 (29)	7483 (30)	2582 (27)	1615 (24)	2809 (43)	2269 (45)	885 (20)	391 (16)	1050 (32)	577 (28)	1194 (41)	790 (36)	218 (21)	158 (20)
**Histology**	Adenocarcinoma	28 795 (76)	19 231 (77)	7316 (75)	4869 (74)	5100 (79)	3979 (78)	NA	NA	2785 (85)	1771 (86)	2693 (93)	2069 (95)	626 (61)	503 (62)
	Signet ring cell carcinoma	6780 (18)	4586 (18)	1850 (19)	1333 (20)	972 (15)	802 (16)	NA	NA	278 (9)	178 (9)	74 (3)	48 (2)	283 (28)	221 (27)
	Other^3^	2254 (6)	1253 (5)	579 (6)	403 (6)	396 (6)	315 (6)	NA	NA	195 (6)	108 (5)	126 (4)	55 (3)	119 (12)	83 (10)
**Differentiation** ^4^	Well	1923 (6)	1487 (6)	261 (4)	211 (4)	60 (12)	479 (11)	–	–	101 (4)	68 (4)	223 (10)	184 (10)	66 (8)	53 (8)
	Moderate	9737 (29)	6753 (29)	1738 (28)	1369 (29)	1729 (31)	1371 (31)	–	–	694 (28)	491 (30)	594 (27)	501 (27)	249 (29)	203 (29)
	Poor/undifferentiated	21 399 (65)	14 968 (65)	4310 (68)	3163 (67)	3158 (57)	2590 (58)	–	–	1659 (68)	1099 (66)	1396 (63)	1139 (62)	554 (64)	447 (64)
**Local invasion** ^5^	Lamina propria/submucosa	11 585 (34)	7070 (29)	1335 (17)	1219 (19)	1426 (23)	1114 (22)	545 (15)	432 (18)	250 (15)	195 (15)	483 (20)	447 (21)	182 (20)	164 (21)
	Muscularis propria/subserosa	14 824 (43)	11 791 (48)	4278 (53)	3509 (55)	2887 (47)	2451 (49)	1950 (53)	1199 (51)	669 (39)	540 (42)	1061 (43)	978 (46)	515 (56)	424 (55)
	Serosa	5071 (15)	4373 (18)	1509 (19)	1283 (20)	1609 (26)	1326 (26)	804 (22)	570 (24)	582 (34)	417 (33)	722 (29)	614 (29)	184 (20)	157 (20)
	Adjacent structures	3032 (9)	1569 (6)	910 (11)	368 (6)	268 (4)	157 (3)	383 (10)	144 (6)	213 (12)	125 (10)	190 (8)	93 (4)	39 (4)	24 (3)
**Positive lymph node** ^6^	0	20 041 (54)	11 631 (47)	4030 (47)	2955 (46)	2787 (46)	2228 (45)	2274 (55)	1275 (51)	1793 (73)	991 (66)	1128 (44)	855 (40)	503 (54)	401 (52)
	1‐6	12 359 (33)	9040 (36)	3414 (40)	2418 (37)	2381 (39)	1908 (38)	1385 (34)	849 (34)	545 (22)	408 (27)	808 (31)	720 (33)	332 (35)	282 (36)
	≥ 7	4507 (12)	4335 (17)	1138 (13)	1081 (17)	948 (16)	856 (17)	464 (11)	361 (15)	126 (5)	94 (6)	638 (25)	578 (27)	103 (11)	95 (12)
**Harvested node no**.		∖	15 ± 13	∖	16 ± 16	∖	NA	∖	18 ± 14	∖	NA	∖	NA	∖	NA
**Resection type** ^7^	Partial/subtotal gastrectomy	∖	16 764 (67)	∖	4564 (69)	∖	NA	∖	860 (62)	∖	NA	∖	NA	∖	NA
	Total/near‐total gastrectomy	∖	5075 (20)	∖	1656 (25)	∖	NA	∖	475 (34)	∖	NA	∖	NA	∖	NA
	Other	∖	3231 (13)	∖	385 (6)	∖	NA	∖	50 (4)	∖	NA	∖	NA	∖	NA
**Resection margin** ^8^	Positive	∖	NA	∖	886 (15)	∖	NA	∖	338 (15)	∖	NA	∖	68 (7)	∖	NA
**Neoadjuvant CHT** ^9^	Yes	∖	NA	∖	2326 (35)	∖	1155 (23)	∖	840 (34)	∖	NA	∖	124 (6)	∖	NA
**Neoadjuvant RT** ^9^	Yes	∖	2509 (10)	∖	183 (3)	∖	204 (4)	∖	188 (8)	∖	NA	∖	61 (3)	∖	NA
**Total/adjuvant CHT** ^9^	Yes	16 872 (45)	11 694 (47)	3176 (33)	1251 (19)	2543 (39)	1646 (32)	NA	NA	614 (19)	453 (22)	813 (28)	620 (29)	177 (17)	134 (17)
**Total/adjuvant RT** ^9^	Yes	12 071 (32)	5766 (23)	810 (8)	215 (3)	1037 (16)	645 (13)	NA	NA	188 (6)	88 (4)	695 (24)	563 (26)	36 (4)	34 (4)

^1^ Categorical data are shown as count (percentage [%]), and numeric data as mean ± standard deviation. Records are complete otherwise specified below. Missing values are shown below.

^2^ The percentages of gastric cardia, fundus/body, and antrum/pylorus cancers are the proportions compared to the total tumor cases of the three locations; “other” includes lesser curvature, greater curvature, and overlapping lesion of stomach and stomach (NOS), and its proportion is relative to the whole cases.

^3^ Cystic/mucinous/serous (excluding signet ring cell), squamous cell, ductal/lobular, complex, unspecified, and epithelial (NOS) neoplasms.

^4^ Unknown differentiation: total patients: the United States, 4770 (13%); the Netherlands, 3436 (35%); Belgium, 941 (15%); Sweden, 3507 (78%); Norway, 804 (25%); Slovenia, 680 (24%); Estonia, 159 (15%); resected patients: the United States, 1862 (7%); the Netherlands, 1862 (28%); Belgium, 656 (13%); Sweden, 1552 (62%); Norway, 399 (19%); Slovenia, 348 (16%); Estonia, 104 (13%).

^5^ Unknown tumor local invasion: total patients: the United States, 3317 (9%); the Netherlands, 1713 (18%); Belgium, 278 (4%); Sweden, 804 (18%); Norway, 1544 (47%); Slovenia, 437 (15%); Estonia, 108 (11%); resected patients: the United States, 267 (1%); the Netherlands, 226 (3%); Belgium, 48 (1%); Sweden, 156 (6%); Norway, 780 (38%); Slovenia, 40 (2%); Estonia, 38 (5%). Invasion of serosa and adjacent structures could not be differentiated from each other in Norway or Slovenia.

^6^ Unknown positive lymph node: total patients: the United States, 922 (2%); the Netherlands, 1163 (12%); Belgium, 352 (5%); Sweden, 363 (8%); Norway, 794 (24%); Slovenia, 319 (11%); Estonia, 90 (9%); resected patients: the United States, 64 (<1%); the Netherlands, 151 (2%); Belgium, 104 (2%); Sweden, 16 (1%); Norway, 564 (27%); Slovenia, 19 (1%); Estonia, 95 (12%).

^7^ Gastrectomy (NOS) or local resection. Available in Sweden since 2010.

^8^ Unkown resection margin for resected nonmetastatic cancer: the Netherlands, 628 (10%); Sweden, 286 (11%); Slovenia, 26 (3%). In Slovenia, margin status was not available before 2009.

^9^ Nonsurgical therapies in the United States and Estonia had low sensitivity, and the counterpart category of “Yes” was “No/unknown.” In Norway and Estonia, neoadjuvant and adjuvant therapies could not be distinguished from each other. Total CHT/RT is for total patients, and (neo)adjuvant CHT/RT for resected patients.

CHT, chemotherapy; RT, radiotherapy; NOS, not otherwise specified; ∖, resection‐specific variables not applicable for total patients; ‐, not shown due to >60% missing values; NA, not available.

### 3.1 Resection trends

**FIGURE 1:**
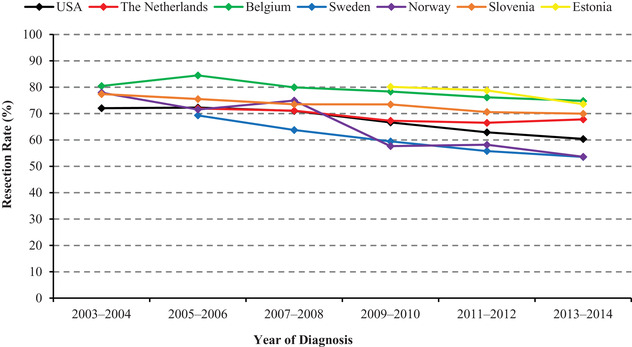
Age‐standardized resection rates for nonmetastatic gastric cancers. In the United States and Norway, the decreasing trends started from as early as the 1980s and the 1960s, respectively (data not shown)

Age‐standardized resection rates decreased over time in all countries (Figure 1). The largest average decreases were observed in Norway (2003‐2004 to 2013‐2014: 78 to 54%; *P_trend _*< .001) and Sweden (2005‐2006 to 2013‐2014: 69 to 54%; *P_trend _*< .001). Moderate decreases were observed in the United States (2003‐2004 to 2013‐2014: 72 to 60%; *P_trend _*< .001) and Estonia (2009‐2010 to 2013‐2014: 80 to 74%; *P_trend _*= .020). The Netherlands (2005‐2006 to 2013‐2014: 72 to 68%; *P_trend _*= .005), Belgium (2003‐2004 to 2013‐2014: 80 to 75%; *P_trend _*< .001), and Slovenia (2003‐2004 to 2013‐2014: 77 to 70%; *P_trend _*= .002) showed the slightest decreases.

When limiting cancers to those without adjacent structure invasion and to those invading beyond submucosa and/or with positive lymph nodes where endoscopic resection was rarely performed, the decreasing resection trends remained in all countries.

### 3.2 Resection trends according to age group and tumor location for nonmetastatic cancers

**FIGURE 2:**
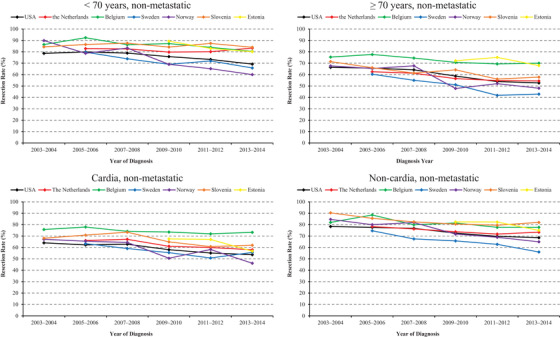
Age‐standardized resection rates for nonmetastatic gastric cancer by age and tumor location

Subgroup analyses of resection trends according to age group and tumor location were further conducted (Figure 2). Resection rates were higher in younger patients, and the decreasing trends were weaker or disappeared in patients <70 years compared to those ≥70 years in the Netherlands (2005‐2006 to 2013‐2014: 83 to 83%, *P_trend _*= .915 vs 63 to 54%, *P_trend _*< .001), Sweden (2005‐2006 to 2013‐2014: 80 to 66%, *P_trend _*= .011 vs.60 to 43%, *P_trend _*< .001), and Slovenia (2003‐2004 to 2013‐2014: 84 to 84%, *P_trend _*= .807 vs 71 to 58%, *P_trend _*= .002). The decreasing trends were stronger in patients <70 years in Norway (2003‐2004 to 2013‐2014: 90 to 60%, *P_trend _*< .001 vs 68 to 48%, *P_trend _*= .001), and Estonia (2009‐2010 to 2013‐2014: 89 to 80%, *P_trend _*= .011 vs.72 to 68%, *P_trend _*= .149). The magnitudes of decrease were similar in both age groups in Belgium (2003‐2004 to 2013‐2014: 86 to 80%, *P_trend _*= .010 vs.75 to 70%, *P_trend _*< .001).

Resection rates for cardia cancers were lower than those for noncardia tumors. The magnitude of decrease was weaker in cardia cancers than noncardia ones in Sweden (2005‐2006 to 2013‐2014: 63 to 56%, *P_trend _*= .008 vs 75 to 56%, *P_trend _*< .001). The trends were only significant in noncardia cancers in Belgium (2003‐2004 to 2013‐2014: 82 to 78%; *P_trend _*= .016), Slovenia (2003‐2004 to 2013‐2014: 90 to 82%; *P_trend _*= .006), and Estonia (2009‐2010 to 2013‐2014: 82 to 75%; *P_trend _*= .035). Similar decreasing magnitudes in cardia and noncardia cancers were observed in the United States (2003‐2004 to 2013‐2014: 64 to 54%, *P_trend _*< .001 vs78 to 69%, *P_trend _*< .001), the Netherlands (2005‐2006 to 2013‐2014: 66 to 58%, *P_trend _*< .001 vs 78 to 73%, *P_trend _*= .016), and Norway (2003‐2004 to 2013‐2014: 67 to 46%, *P_trend _*= .001 vs 85 to 65%, *P_trend _*< .001).

### 3.3 Recent resection rates for GC by age group and tumor location

**FIGURE 3:**
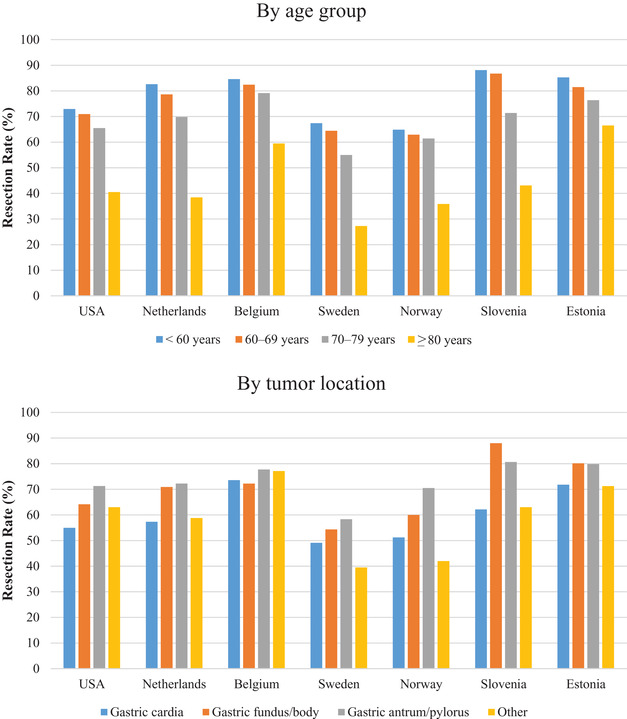
Resection rates for nonmetastatic gastric cancers by age group and tumor location in 2010 or later

We limited the patients to those diagnosed in 2010 or later, a recent period when all countries had data, to calculate the resection rates according to age group and tumor location (Figure 3). Resection rates decreased with increasing ages in all countries. The rates were markedly lower in patients ≥80 years (27% [Sweden] to 66% [Estonia]) compared to the other age groups (<60 years: 65% [Norway] to 88% [Slovenia]; 60‐69 years: 63% [Norway] to 87% [Slovenia]; 70‐79 years: 55% [Sweden] to 79% [Belgium]), with large variations across countries. In most countries, resection rates were lower for cardia cancers (49% [Sweden] to 74% [Belgium]) than for fundus/body (54% [Sweden] to 88% [Slovenia]) or pylorus/antrum cancers (58% [Sweden] to 81% [Slovenia]).

### 3.4 Factors associated with resection

We further investigated variables associated with resection in each country using multivariable‐adjusted log‐binomial models (Table 3), which further supported the decreasing resection rates (PR per year = 0.97 [Sweden, Norway, and Estonia] to 0.995 [the Netherlands] across countries).

**TABLE 3:** Association of demographic and clinical parameters with resection for gastric cancer without distant metastasis using multivariable‐adjusted log‐binomial regression

		The US	The Netherlands	Belgium	Sweden	Norway	Slovenia	Estonia
**Variable**	Category	PR (95% CI)^1^	PR (95% CI)^1^	PR (95% CI)^1^	PR (95% CI)^1^	PR (95% CI)^1^	PR (95% CI)^1^	PR (95% CI)^1^
Year of diagnosis	Per 1 year	**0.98** (0.98‐0.99)	**0.995** (0.99‐1.00)	**0.99** (0.99‐0.99)	**0.97** (0.96‐0.98)	**0.97**(0.97‐0.98)	**0.99** (0.99‐1.00)	**0.97** (0.96‐0.99)
Sex	Female vs male	1.00 (0.99‐1.01)	0.99 (0.96‐1.01)	1.00 (0.97‐1.02)	1.06 (0.96‐1.06)	1.01 (0.96‐1.05)	1.01 (0.98‐1.04)	1.04 (0.98‐1.10)
Age group	60‐69 years	0.99 (0.98‐1.01)	**0.97** (0.94‐0.99)	0.99 (0.96‐1.02)	0.99 (0.94‐1.05)	1.00 (0.97‐1.04)	0.97 (0.94‐1.00)	0.94 (0.87‐1.01)
<60 years as reference	70‐79 years	**0.93** (0.91‐0.94)	**0.86** (0.84‐0.89)	**0.94** (0.91‐0.97)	**0.88** (0.83‐0.93)	**0.95** (0.91‐1.00)	**0.93** (0.89‐0.96)	**0.91** (0.85‐0.98)
	≥80 years	**0.63** (0.62‐0.65)	**0.53** (0.50‐0.55)	**0.73** (0.70‐0.76)	**0.49** (0.45‐0.54)	**0.65** (0.61‐0.70)	**0.73** (0.69‐0.77)	**0.79** (0.72‐0.87)
Tumor location	Gastric fundus/body	**1.18** (1.15‐1.20)	**1.17** (1.13‐1.22)	**1.06** (1.01‐1.12)	**1.15** (1.08‐1.23)	**1.21**(1.11‐1.31)	**1.18** (1.12‐1.24)	1.09 (0.98‐1.22)
Gastric cardia as reference	Gastric antrum/pylorus	**1.30** (1.28‐1.32)	**1.23** (1.19‐1.27)	**1.11** (1.07‐1.14)	**1.21** (1.14‐1.28)	**1.34** (1.25‐1.43)	**1.14** (1.09‐1.20)	1.10 (0.99‐1.22)
	Other^2^	**1.18** (1.16‐1.20)	1.03 (0.99‐1.07)	**1.08** (1.05‐1.12)	**0.91** (0.83‐0.99)	1.03 (0.95‐1.11)	1.01 (0.96‐1.07)	1.00 (0.89‐1.12)
Tumor histology	SRC vs non‐SRC	**0.96** (0.94‐0.98)	0.99 (0.97‐1.02)	1.01 (0.99‐1.04)	–	0.98 (0.91‐1.06)	0.90 (0.80‐1.01)	0.96 (0.90‐1.02)
Adjacent structure invasion	Yes vs no	**0.68** (0.66‐0.70)	**0.71** (0.68‐0.74)	**0.72** (0.66‐0.79)	**0.57** (0.50‐0.65)	**0.80** (0.71‐0.89)	**0.68** (0.61‐0.75)	**0.78** (0.64‐0.96)

^1^ Prevalence ratios and 95% confidence intervals for resection versus nonresection were calculated using multivariable‐adjusted log‐binomial regression models adjusting for year of diagnosis, sex, age group, tumor location, and histology. For the association with adjacent structure invasion, this factor was additionally added into the main model. Previous cancer was available and also adjusted for in the United States, the Netherlands, and Belgium. All models converged. PRs shown in bold are statistically significant.

^2^ Lesser curvature, greater curvature, and overlapping lesion of stomach, and stomach (not otherwise specified).

CI, confidence interval; PR, prevalence ratio; SRC, signet ring cell carcinoma; ‐, not available; ∖, not estimable.

While resection was not significantly associated with sex, it was less frequently performed with older age and for cardia cancer in all countries. Specifically, compared to patients <60 years, PRs for resection in patients aged 70‐79 and ≥80 years were 0.86 (the Netherlands) to 0.95 (Norway) and 0.49 (Sweden) to 0.79 (Estonia), respectively. In all countries except Estonia where associations with location were insignificant, resection rates of fundus/body and antrum/pylorus cancers were higher than those of cardia cancers, with PRs ranging from 1.06 (Belgium) to 1.21 (Norway) and from 1.11 (Belgium) to 1.34 (Norway), respectively. Resection was less often conducted for signet ring cell carcinomas (SRCs) in the United States. Adjacent structure invasion was associated with less frequent resection in all countries (PR = 0.57 [Sweden] to 0.80 [Norway]).

Associations of resection with further variables available in certain countries are shown in Table 4. Management in academic hospitals was associated with more frequent resection in the Netherlands, Belgium, and Sweden. In the Netherlands and Sweden, a smaller hospital volume was associated with less frequent resection. In the United States, resection was more frequently performed for smaller tumors. With higher ECOG‐PS and ASA scores, resection was much less often performed. Cardiac disease, vascular disease, and pulmonary disease were associated with less frequent resection. More than two comorbidities were associated with 9% reduced resection rates in Eindhoven. The decreasing resection trends over time remained after adjusting for these factors.

**TABLE 4:** Association of hospital type, hospital volume, tumor size, performance status, and comorbidities with resection in nonmetastatic gastric cancer in registries with available information using multivariable‐adjusted log‐binomial regression

		The US		The Netherlands		Belgium		Sweden	
Variable	Category	n	PR (95% CI)^1^	N	PR (95% CI)	n	PR (95% CI)	n	PR (95% CI)
Hospital type	Nonacademic	–	–	7857	1.00 (reference)	3906	1.00 (reference)	2515	1.00 (reference)
	Academic	–	–	1875	**1.13** (1.11‐1.16)	2510	**1.05** (1.03‐1.07)	1971	**1.11** (1.06‐1.16)
Hospital volume (resections/year)	<10	–	–	1232	**0.93** (0.90‐0.96)	–	–	872	**0.92** (0.87‐0.96)
	10‐20	–	–	1374	**0.94** (0.91‐0.97)	–	–	931	0.97 (0.94‐1.01)
	≥20	–	–	1000	1.00 (reference)	–	–	1373	1.00 (reference)
Tumor size (cm)	<2	1694	**1.04** (1.03‐1.05)	–	–	–	–	–	–
	2‐4	2747	**1.02** (1.01‐1.03)	–	–	–	–	–	–
	≥4	5056	1.00 (reference)	–	–	–	–	–	–
ECOG score	0‐1	–	–	–	–	4285	1.00 (reference)	3194	1.00 (reference)
	2	–	–	–	–	510	**0.87** (0.81‐0.93)	763	**0.51** (0.45‐0.57)
	≥3	–	–	–	–	159	**0.48** (0.39‐0.60)	287	**0.15** (0.10‐0.22)
ASA score	1‐2	–	–	–	–	–	–	2949	1.00 (reference)
	3	–	–	–	–	–	–	1166	**0.74** (0.68‐0.79)
	≥4	–	–	–	–	–	–	236	**0.31** (0.24‐0.42)
Comorbidity	Cardiac disease	–	–	615/1437	**0.93** (0.87‐1.00)	3405/3063	0.99 (0.96‐1.01)	–	–
	Vascular disease	–	–	349/1703	**0.89** (0.81‐0.98)	–	–	–	–
	Hypertension	–	–	588/1464	1.00 (0.94‐1.06)	–	–	–	–
	Diabetes	–	–	336/1716	0.94 (0.86‐1.02)	980/5488	0.97 (0.93‐1.00)	–	–
	Pulmonary disease	–	–	255/1797	0.95 (0.86‐1.05)	370/6098	**0.94** (0.88‐1.00)	–	–
Comorbidity no.	0	–	–	609	1.00 (reference)	–	–	–	–
	1	–	–	548	1.00 (0.95‐1.06)	–	–	–	–
	≥2	–	–	895	**0.91** (0.85‐0.98)	–	–	–	–

^1^ Prevalence ratios and 95% confidence intervals for associations of hospital type, hospital volume, tumor size, ECOG score, ASA score, and comorbidity with resection versus nonresection were calculated by adding these variables one by one into the main multivariable‐adjusted log‐binomial regression models adjusting for year of diagnosis, sex, age group, tumor location, and histology. The reference categories for each comorbidity were those without the corresponding comorbidity. Previous cancer was available and also adjusted for in the United States, the Netherlands, and Belgium. All models converged. Statistically significant prevalence ratios are shown in bold. Numbers for comorbidities are shown for with/without the respective comorbidity.

ASA, American Society of Anesthesiologists; CI, confidence interval; ECOG, Eastern Cooperative Oncology Group PR, prevalence ratio; ‐, not available.

### 3.5 Subgroup analyses regarding the association of resection with year of diagnosis

Subgroup analyses on the association of resection with year of diagnosis were performed according to age, tumor location, histology, and invasion (Table 5). We selected 70 years as the age cutoff, considering the findings that compared to patients aged <60 years, those aged 70‐79 and ≥80 years had significantly lower resection rates in most countries, while the resection rates for those aged 60‐69 years were mostly not significantly different (Table 3), and that patients aged ≥70 years comprised nearly half of the total patients (51‐62%) (Table 1).While association patterns in subgroups were mostly similar to the overall ones, some interesting differences were observed.

**TABLE 5:** Association of year of diagnosis (as continuous) with resection for gastric cancer without distant metastasis using multivariable‐adjusted log‐binomial regression in various subgroups

Variable		The US	The Netherlands	Belgium	Sweden	Norway	Slovenia	Estonia
<70 years	Resected/total no.	13 162/17 537	3238/3979	2301/2664	1226/1757	909/1245	1120/1304	416/496
	PR per 1 year (95% CI)^1^	**0.99** (0.99‐0.99)	1.00 (0.99‐1.00)	**0.99** (0.98‐0.99)	**0.98** (0.97‐0.99)	**0.97**(0.96‐0.98)	1.00 (0.99‐1.01)	**0.97** (0.95‐0.99)
≥70 years	Resected/total no.	11 908/20 292	3367/5766	2795/3804	1275/2729	1148/2013	1052/1589	391/532
	PR per 1 year (95% CI)^1^	**0.98** (0.98‐0.98)	**0.99** (0.98‐0.99)	**0.99** (0.99‐1.00)	**0.96** (0.95‐0.97)	**0.98** (0.97‐0.98)	**0.98** (0.98‐0.99)	**0.96** (0.93‐0.99)
Cardia	Resected/total no.	7387/12 731	1622/2630	1520/2024	737/1387	480/857	314/461	70/96
	PR per 1 year (95% CI)^1^	**0.99** (0.98‐0.99)	**0.98** (0.97‐0.99)	0.99 (0.99‐1.00)	**0.97** (0.96‐0.99)	**0.96** (0.95‐0.97)	0.99 (0.97‐1.01)	0.95 (0.89‐1.03)
Noncardia	Resected/total no.	10 200/14 039	3368/4533	1307/1635	1373/2215	1000/1351	1068/1238	579/714
	PR per 1 year (95% CI)^1^	**0.99** (0.98‐0.99)	1.00 (0.99‐1.00)	**0.99** (0.98‐1.00)	**0.97** (0.96‐0.98)	**0.98** (0.97‐0.99)	0.99 (0.99‐1.00)	**0.98** (0.96‐1.00)
Signet ring cell carcinoma	Resected/total no.	4586/6780	1333/1850	802/972	–	178/278	48/74	221/283
	PR per 1 year (95% CI)^1^	**0.98** (0.98‐0.98)	**0.99** (0.98‐1.00)	**0.99** (0.98‐1.00)	–	0.99 (0.97‐1.01)	/	0.97 (0.94‐1.00)
Without adjacent structure invasion	Resected/total no.	23 234/31 480	6011/7122	4890/5922	2201/3299	1152/1501	2039/2266	745/881
	PR per 1 year (95% CI)^1^	**0.99** (0.99‐0.99)	**0.99** (0.99‐0.99)	**0.99** (0.99‐0.99)	**0.97** (0.96‐0.98)	∖	1.00 (0.99‐1.00)	**0.98** (0.96‐0.99)
With adjacent structure invasion	Resected/total no.	1569/3032	368/910	157/268	144/383	125/213	93/190	24/39
	PR per 1 year (95% CI)^1^	**0.99** (0.98‐1.00)	**1.07** (1.04‐1.09)	1.01 (0.97‐1.05)	**0.91** (0.87‐0.95)	1.00 (0.98‐1.02)	1.06 (1.00‐1.12)	/
With tumor invasion beyond submucosa	Resected/total no.	18 842/24 996	5407/7256	4132/5059	2002/3347	1112/1527	1760/2048	633/768
and/or with positive lymph nodes	PR per 1 year (95% CI)^1^	**0.99** (0.98‐0.99)	**0.99** (0.99‐1.00)	**0.99** (0.99‐1.00)	**0.97** (0.96‐0.97)	∖	1.00 (0.99‐1.00)	**0.97** (0.96‐0.99)

^1^ Prevalence ratios and 95% confidence intervals for association of year of diagnosis (as continuous) with resection versus nonresection were calculated using multivariable‐adjusted log‐binomial regression models adjusting for sex, age group, tumor location, and histology. Previous cancer was available and also adjusted for in the United States, the Netherlands, and Belgium. All models converged. Results were not shown for countries with <40 resected and/or <80 total cases. PRs shown in bold are statistically significant.

PR, prevalence ratio; CI, confidence interval; ‐, not available; ∖, not shown for non‐metastatic cases in Norway due to too high proportions of missing values for both tumor local invasion (47%) and positive lymph node (24%); /, not shown due to small case number (<40 resected and/or <80 total cases).

Compared to patients <70 years, the decreasing trends were stronger in those ≥70 years in the United States, the Netherlands, Sweden, and Slovenia. Compared to noncardia cancers, the decreasing trends were stronger for cardia cancers in the Netherlands and Norway. In SRCs, in cancers without adjacent structure invasion, and in cancers invading beyond submucosa or having positive lymph nodes, association patterns and strengths were mostly similar with those for total cancers. For cancers invading adjacent structure, resection rates increased in the Netherlands and more strongly decreased in Sweden, and trends became insignificant in Belgium, Norway, and Slovenia.

## 4 DISCUSSION

To our knowledge, this is the largest international population‐based report on patient and tumor characteristics, treatment trends, and treatment‐associated factors for nonmetastatic GC across Europe and the United States. Our study revealed *large variations* in treatment patterns across countries. Somewhat unexpectedly, resection rates decreased for nonmetastatic cancers, for which resection remains the only curative treatment. The decreasing trend was consistently seen in various subgroups. Furthermore, several tumor and patient characteristics associated with resection were revealed.

The observed decreasing trends are consistent with some previous studies from the United States and the Netherlands. In the United States during 1983‐2002, resection rates declined by 6% units in all stages, and by even 20% units in local stages.^14^, ^15^ Using the US Nationwide Inpatient Sample in 1988‐2000, gastric resection rate showed a 20% decline.^16^ In the Netherlands, resection rates for stage I‐III noncardia cancer decreased from 71% in 1989‐1992 to 62% in 2005‐2008, while rates for cardia cancer remained relatively stable during that period.^9^ Resection trends in the other European countries have been rarely reported.

In Western countries, there is consensus that medically fit patients with nonmetastatic resectable GC should undergo standardized resection in specialized, high‐volume centers.^17^, ^18^, ^19^ While GC surgery has shown some trends of centralization, the degree and the initiation time vary across countries. We found that proportions of patients managed and of resections performed in academic hospitals increased moderately in the Netherlands (2005‐2014: 14‐22% and 17‐34%) and Belgium (2004‐2013: 38‐43% and 40‐47%), and strongly in Sweden (2006‐2016: 34‐70% and 38‐84%). In the United States, proportions of gastrectomy performed at centers with ≥9 yearly resections increased from 43% in 1988‐1989 to 48% in 1999‐2000.^16^ We found that the proportions of patients managed (27‐29%) and of resections performed (33‐33%) in hospitals with ≥20 annual gastric/esophageal resections remained relatively stable during 2005‐2014 in the Netherlands, where centralization of GC surgery has essentially been imposed since 2012 only.^20^, ^21^ Proportions of resections done in hospitals with ≥20 yearly resections increased moderately in Belgium (2004‐2013: 18‐28%). In Sweden, proportions of patients treated (30‐72%) and of resections (32‐68%) performed in hospitals with ≥20 yearly resections increased strongly in 2006‐2016.

We found that GC was most commonly diagnosed in patients ≥70 years and at stomach cardia. For elderly patients who are more commonly frail the resection‐upfront approach might be suboptimal unless specifically tailored.^22^ Geriatric assessment would be helpful before initiating treatment for older patients. However, GC patients were getting increasingly younger in the investigated period. Cardia cancer may be more surgically challenging.^23^ While the recently increasing incidence of cardia cancer potentially impedes resection,^5^, ^24^ resection rates for noncardia cancer were also decreasing. Cancers invading adjacent structures had lower R0 resection rates, which potentially bars resection. The different patterns and strengths of associations of resection with patient and tumor characteristics across countries highlight the variation in clinical practice and the need for standardization.

We have observed increasing clear‐margin (R0) resection rates among all resections for nonmetastatic cancer in the Netherlands (2005‐2014: 83‐88%) and Sweden (2006‐2016: 83‐92%). Furthermore, proportions of resections with ≥15 examined lymph nodes for nonmetastatic disease increased in the United States (2004‐2014: 36‐51%), the Netherlands (2005‐2014: 32‐67%), and Sweden (2006‐2016: 42‐82%). While these trends could partly reflect the surgical advances, they might also indicate the increasingly stricter selection criteria of resection candidates. While resection rates decreased, the indicated higher success rates and better quality of resection could contribute to better results for resected patients.

Following the pivotal MAGIC trial (2006),^25^ perioperative therapy is recommended as standard of care for most resectable GC planned for resection throughout many parts of Europe, and is increasingly favored over adjuvant treatment.^25^, ^26^, ^27^, ^28^ While the preoperative approach might enhance resectability by down‐staging tumor, it also allows substantial time for further growth of advanced cancers or metastases, which potentially impeded the application of resection. Greater access to and wider use of chemotherapy and/or radiotherapy and presurgical chemotherapy‐associated toxicity might also preclude some patients from receiving further resection.^29^, ^30^

Proper patient selection for treatment is paramount. Physician recommendation and expertise, and patient preference and adherence importantly impact treatment choice. In patients with unresected nonmetastatic GC in SEER‐18, the proportion of those recommended for surgery decreased from 12% in 2004 to 11% in 2014. The aggressive nature of GC and historically poor outcomes even in the setting of operable disease should be discussed with patients before treatment. Patient nutrition and psychosocial statuses, organ function, medical history, tolerability, therapeutic burden especially cost, potential benefit from resection, postoperative morbidity and mortality, and quality of life should also be factored into treatment decisions.

This study was limited by its observational nature. Some variables were not recorded in certain countries, and the quality of registration might vary. *Endoscopic resection was recorded and clearly differentiated from surgical resection only in the Netherlands and Sweden. To maintain the consistency of definition and analysis across countries, the term “resection” rather than “surgery” was used in our study. Endoscopic resection is in essence a type of and belongs to resection, like the case of surgical resection. Sensitivity analyses by limiting patients to those who are clearly not appropriate candidates for endoscopic resection revealed similar trends*. While variables included in the main models were complete, some variables were not included in modeling due to the relatively high proportions of missing values (e.g., differentiation). Proportions of unknown metastasis were particularly high in Belgium (22%) compared to the other countries (4‐10%). We did not pool or compare data between countries, considering the potential heterogeneity, but analyzed, presented, and interpreted data for each country separately. It is noteworthy that the proportion of cardia cancer was very low in Slovenia (27%) and Estonia (12%), and SRC was very often diagnosed in Estonia (28%). While this could be partly explained by differences in dietary and obesity patterns and the prevalence of *Helicobacter pylori* infection, potential variation in clinical and registry practice might also play a role which underlines the importance of further standardization. The reasons for the observed decreasing resection trends were not totally clear. Further studies are needed to explore factors accounting for the observed decreasing resection rates. The investigated time periods were not totally identical. Nevertheless, they mostly covered the period 2003/2004‐2013/2014, and year of diagnosis was adjusted for in all multivariable models. Finally, our analyses were restricted to the United States and European countries, and the results may not be generalized to other parts of the world, especially countries in East Asia where the incidence rates of GC are high.

Notably, the largest sample size ever investigated, uniformly defined variables across nationwide population‐based registries from multiple countries, careful case selection and quality control, and standardized statistical methods enabled this report to show important informative results regarding treatment for GC that warrants clinicians’ and policymakers’ great attention.

In conclusion, nonmetastatic GCs were less frequently resected in Europe and the United States in the early 21st century. Resection rates varied greatly across countries, and appeared not to be optimal. Various variables associated with resection were revealed. Our findings can help to identify differences and possibly modifiable places in clinical practice and provide important novel references for designing effective population‐based GC management strategies, of potential important relevance for guiding adequate health policymaking and resource allocation.

## AUTHORS’ CONTRIBUTIONS

Guarantor of the article: Hermann Brenner, MD, MPH. Conception or design: Huang L, Jansen L, Schrotz‐King P, and Brenner H.

Acquisition, analysis, or interpretation of data: Huang L, Jansen L, Balavarca Y, VerhoevenR, Ruurda J, Van EyckenL, De SchutterH, Johasson J, Lindblad M, Johannesen T, Zadnik V, Zagar T, Mägi M, Bastiaannet E, Lagarde S, van de Velde C, Schrotz‐King P, and Brenner H. Drafting of the manuscript: Huang L. Critical revision of the manuscript for important intellectual content: Jansen L, Balavarca Y, VerhoevenR, Ruurda J, Van EyckenL, De SchutterH, Johasson J, Lindblad M, Johannesen T, Zadnik V, Zagar T, Mägi M, Bastiaannet E, Lagarde S, van de Velde C, Schrotz‐King P, Brenner H. Statistical analysis: Huang L, Balavarca Y. Administrative, technical, or material support: van de Velde C and Brenner H.

All authors have given final approval of the manuscript for submission and publication.

## ETHICS APPROVAL

This study was approved by the Ethics Committee of the Medical Faculty Heidelberg. Consent was not obtained for this observational, population‐based, and registry‐based study. The presented secondary data are anonymous without any risk of identification, and no individual patient data were reported.

## AVAILABILITY OF DATA AND MATERIAL

The Surveillance, Epidemiology, and End Results (SEER) Program data are available upon reasonable request and with permission of the registry. The other data that support the findings of this study are available from each participating registry but restrictions apply to the availability of these data, which were used under license for the current study, and so are not publicly available.

## CONFLICT OF INTEREST

The authors declare that they have no conflict of interest.

## Supporting information

SUPPORTING INFORMATION
