# Supplementary material for: Decreasing resection rates for nonmetastatic gastric cancer in Europe and the United States
Source: Clin Transl Med. 2020 Oct 8;10(6):e203. doi: 10.1002/ctm2.203 (PMC7586997; doi:10.1002/ctm2.203)
Supplement: Supplementary file 1 — SUPPORTING INFORMATION [file CTM2-10-e203-s001.docx]

**Supplementary Materials**

**Supplementary Results**

**Table S1.** Selection of contacted national population-based cancer registries in Europe

**Table S2.** Inclusion and exclusion codes according to International Classification of Diseases for Oncology, Third Edition

**Table S3.** Overall resection rates of all patients and patients after exclusion of non-pathologically diagnosed/eligible cases, those with unknown metastasis status, and both

**Supplementary Results**

***Characteristics of overall and resected non-metastatic gastric cancer (GC) patients***

Among overall patients with non-metastatic disease, most were males (55%-66%), and the mean ages were 68-72 years, with patients ≥70 years comprising the majority (51%-62%). Gastric cardia was the most common cancer site (37%-55%), except in Slovenia (27%) and Estonia (12%). Most tumors were adenocarcinomas followed by signet-ring cell carcinomas (SRCs). Except Slovenia with a particularly low proportion of reported SRCs (3%) and Estonia with a particularly high proportion of SRCs (28%), the proportions of non-SRC adenocarcinomas were 75%-85% in the other countries. Most cancers were poorly-/undifferentiated (57%-68%). Approximately half of the cancers invaded muscularis propria/subserosa (39%-56%), and did not involve lymph nodes (44%-73%). Only 4%-12% of cancers invaded adjacent structures. Over the studied period, resection rates ranged from 56% (Sweden) to 79% (Belgium and Slovenia). In the investigated countries except the US and Estonia where non-surgical therapies had low sensitivity, chemotherapy was administered to 19% (Norway) to 39% (Belgium) of the patients, and radiotherapy was less often applied (6% (Norway) to 24% (Slovenia)).

Resected patients were younger (mean ages, 67-70 years), with smaller proportions of patients ≥70 years (47%-56%). Cardia cancers comprised smaller proportions (11%-54%), and smaller proportions of cancers invaded adjacent structures (3%-10%) or spared lymph nodes (40%-66%). On average, 15-18 lymph nodes were harvested. Partial/subtotal gastrectomy was the most common resection type (62%-69%), and proportions of positive resection margin were 15% in the Netherlands and Sweden and 7% in Slovenia. In countries with available information of high sensitivity, neoadjuvant chemotherapy was administered for 6% (Slovenia) to 35% of the patients (the Netherlands), while neoadjuvant radiotherapy was rarely administered (3% (the Netherlands and Slovenia) to 8% (Sweden)); adjuvant chemotherapy was used for 19%-32% of patients, while adjuvant radiotherapy was less frequently administered (3%-26%).

**Table S1.** Selection of contacted national population-based cancer registries in Europe^1^

| Country of contacted registry | Included | Comment if not included |
| --- | --- | --- |
| *Northern Europe* |  |  |
| Finland | No | Surgical treatment not validated |
| Sweden | Yes | Included |
| Norway | Yes | Included |
| Iceland | No | No national population-based data on treatment |
| Denmark | No | Required variables not readily prepared |
| *Western Europe* |  |  |
| The UK | No | No ready-to-use national population-based data on treatment or TNM stage |
| Ireland | No | No further response after initial contact |
| The Netherlands | Yes | Included |
| Belgium | Yes | Included |
| *Southern Europe* |  |  |
| Bulgaria | No | No national population-based data on treatment |
| Serbia | No | No response |
| Slovenia | Yes | Included |
| Croatia | No | No national population-based data on surgical treatment |
| *Eastern Europe* |  |  |
| Estonia | Yes | Included |
| Latvia | No | No national population-based data on treatment |
| Lithuania | No | No response |
| Ukraine | No | Insufficient resources for data collection |
| Slovakia | No | No response |
| *Central Europe* |  |  |
| Poland | No | No response |
| Czech Republic | No | No national population-based data on treatment |
| Austria | No | No national population-based data on treatment |

^1^For the other countries and regions in Europe not listed in this table, no corresponding national population-based registries with required variables (e.g., TNM stage and treatment) were found through careful search.

**Table S2.** Inclusion and exclusion codes according to International Classification of Diseases for Oncology, Third Edition^1^

| **Category** |  | **Code** |
| --- | --- | --- |
| **Topology** | Inclusion | C16, C16.0, C16.1, C16.2, C16.3, C16.4, C16.5, C16.6, C16.8, C16.9 |
|  | Exclusion | - |
| **Morphology** | Inclusion^2^ | 8000-8009 (unspecified neoplasms), 8010-8049 (epithelial neoplasms, NOS), 8050-8089 (squamous cell neoplasms), 8140-8389 (adenomas and adenocarcinomas), 8440-8499 (cystic, mucinous and serous neoplasms), 8500-8549 (ductal and lobular neoplasms), 8550-8559 (acinar cell neoplasms), 8560-8579 (complex epithelial neoplasms) |
|  | Exclusion | 8013, 8152, 8153, 8156, 8160, 8170, 8240-8243, 8246, 8249, 8252, 8390, 8590, 8680, 8700, 8711, 8720, 8800-8805, 8810, 8811, 8830, 8840, 8850-8852, 8858, 8890, 8891, 8895-8897, 8900, 8902, 8910, 8912, 8920, 8930, 8931, 8935, 8936, 8960, 9040, 9041, 9064, 9071, 9080, 9090, 9100, 9120, 9364, 9380, 9490, 9500, 9540, 9560, 9580 |
| **Behavior** | Inclusion | 3 |
|  | Exclusion | 0, 2 |

^1^http://codes.iarc.fr/

^2^Based on [Surveillance, Epidemiology, and End Results Program](http://seer.cancer.gov/) broad groupings.

**Table S3.** Overall resection rates of all patients and patients after exclusion of non-pathologically diagnosed/eligible cases, those with unknown metastasis status, and both

| **Patients** | **The US** | **The Netherlands** | **Belgium** | **Sweden** | **Norway** | **Slovenia** | **Estonia** |
| --- | --- | --- | --- | --- | --- | --- | --- |
|  | Resection rate (%) | Resection rate (%) | Resection rate (%) | Resection rate (%) | Resection rate (%) | Resection rate (%) | Resection rate (%) |
| All patients | 46 | 45 | 51 | 36 | 43 | 51 | 50 |
| After exclusion of non-pathologically diagnosed/eligible cases | 44 | 46 | 50 | 36 | 44 | 52 | 52 |
| After exclusion of those with unknown metastasis status | 49 | 47 | 61 | 38 | 45 | 54 | 56 |
| After exclusion of both | 47 | 47 | 61 | 38 | 45 | 55 | 57 |
| Patients finally included | 46 | 46 | 61 | 38 | 45 | 55 | 57 |
